# Clinical aspects of envenomation by a green pit viper (*Bothrops bilineatus*), an arboreal snake

**DOI:** 10.1590/0037-8682-0178-2026

**Published:** 2026-07-17

**Authors:** César Mancilha Carvalho Pedigone, Marcela Thiemi Andrade Korogi, Vidal Haddad

**Affiliations:** 1 Instituto Flor da Floresta - Ni Huá, Jordão, AC, Brasil.; 2 Universidade Estadual Paulista, Faculdade de Medicina de Botucatu, Botucatu, SP, Brasil.

Dear Editor:

The green lancehead viper, also known as the two-striped forest-pitviper, parrotsnake, golden droplet, or patioba surucucu, is a viperid snake found in the Amazon rainforest and the Atlantic Forest. It is sometimes identified as *Bothriopsis bilineata*, but most authors classify it as *Bothrops bilineatus* or *Bothrops bilineata*. Currently, two subspecies have been identified: *Bothrops bilineatus bilineatus* (Wied-Neuwied, 1825) in the Atlantic Forest and *Bothrops bilineatus smaragdinus* (Hoge, 1966) in the Amazon region^1^ ^-^ ^3^.

Although official data are lacking, green lancehead vipers are considered as a significant cause of envenomation in the Amazon. This snake exhibits arboreal behavior, preferring areas near bodies of water, and a peculiar aspect of the bites in humans is that they occur in elevated body areas (hands, arms, and face), due to the snake's position in trees^1^ ^,^ ^3^ ^,^ ^4^. 

The venom of these snakes has an action similar to that of other snakes of the genus *Bothrops* (jararacas, pit vipers, lancehead vipers), acting locally with a proteolytic effect, which causes intense tissue destruction (presenting as significant pain, edema, erythema, hemorrhagic blisters, and necrosis) in addition to coagulant/hemorrhagic phenomena that manifest as petechiae, hematomas, and hemorrhagic suffusions locally and systemically, including various organs, causing hematemesis, hematuria, gingival bleeding, and possible hemorrhages in the central nervous system, which may lead to loss of consciousness, coma, and death. Incoagulable blood can be detected through a simple coagulation time test, an important predictive factor for determining the doses of antivenom (antibothropic serum (pentavalent or antibothropic-lachesis) to be administered^1^ ^,^ ^5^. There is a report of one death in the literature^6^.

**FIGURE 1:**
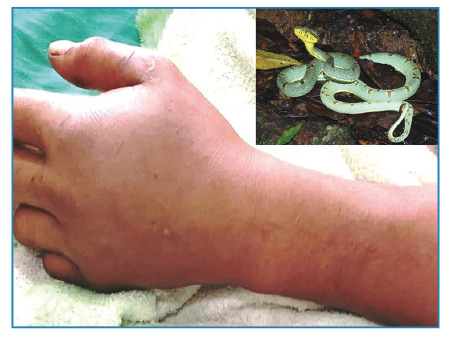
*Bothrops bilineatus* (photo by Marco Antônio de Freitas) and the hand of the envenomation victim, showing a discreet bite mark and bruises on the hand and forearm.

A 25-year-old male patient with a history of alcoholism and smoking was bitten by a green lancehead viper while pushing away tree branches with his left hand in a remote area of the Amazon. He killed the snake and brought it to the hospital in the town of Jordão, in the state of Acre, Brazil, approximately 6 h after the bite. The snake was identified by hospital staff due to the characteristic appearance of the species. During this time, he noticed intense edema and erythema on his hand and the appearance of "dark spots", and he experienced nausea and vomiting (Figure 1). Due to the presence of significant local symptoms, but with normal coagulation time, he was treated with 6 ampoules of antibothropic serum, developing an allergic reaction of the urticaria type that resolved with hydrocortisone and dexchlorpheniramine. In the following two days, the local clinical manifestations continued and the coagulation time increased (20 minutes, with a local reference value of 4-9 minutes) and six more doses were applied with care, considering the prior allergic reaction. After gradual improvement, he was discharged from the hospital after 6 days.

Despite the patient's favorable outcome, the case shows that bites of these snakes shares the characteristics indicated in previous reports, presenting the potential for severe envenomation from snakes of the genus Bothrops, but affecting body areas less common in other occurrences involving venomous snakes in the country. This underscores the need for increasing awareness and providing guidance, particularly for forest workers. 
